# Study of the cervical canal microbiome and microbiocenosis in reproductive-age women with squamous intraepithelial lesion

**DOI:** 10.3389/fmicb.2025.1630092

**Published:** 2025-09-02

**Authors:** Anastasiya Peremykina, Valery Cheranev, Alexey Y. Shilyaev, Andrey Krivoy, Zhanna Repinskaia, Dmitriy Korostin, Denis Rebrikov

**Affiliations:** ^1^Department of Outpatient Clinical Research Development, National Medical Research Center for Obstetrics, Gynecology and Perinatology Named After Academician V.I. Kulakov, Ministry of Healthcare of the Russian Federation, Moscow, Russia; ^2^Center for Precision Genome Editing and Genetic Technologies for Biomedicine, Pirogov Medical University, Moscow, Russia

**Keywords:** SIL, CC, NGS, HPV, bacterial vaginosis, aerobic vaginitis, lactobacilli, microbiome

## Abstract

**Introduction:**

The study of the cervicovaginal microbiome is a critical area of research in medical science. According to scientific data, microorganisms inhabiting the lower female genital tract may influence susceptibility to and persistence of human papillomavirus (HPV), as well as the development and progression of squamous intraepithelial lesions (SIL) toward cervical cancer (CC).

**Methods:**

The study included 67 patients with a histological diagnosis of low-grade squamous intraepithelial lesion (LSIL) and 85 patients with high-grade squamous intraepithelial lesion (HSIL). The cervical canal microbiome of patients with LSIL (*n* = 31) and HSIL (*n* = 45) was examined using the 16S rRNA gene sequencing method, while the microbiocenosis of the remaining patients with LSIL (*n* = 36) and HSIL (*n* = 40) was analyzed using PCR-RT.

**Results:**

There are patients with HSIL on average older than patients with LSIL about 3 years. HPV 16 was found to be the most common virus type in patients with SIL. When determining of 16S rRNA genes of prokaryotic microorganisms in women of reproductive age with SIL by the new generation sequencing, it turned out that the cervical canal microbiome is inhabited by many atypical representatives (soil, aquatic and aerobacteria). In addition to the genera *Lactobacillus, Streptococcus, Staphylococcus, Gardnerella, Ureaplasma, Prevotella, Sneathia, Fusobacterium, Veillonella, Megasphaera, Dialister, Enterococcus, Escherichia/Shigella, Fannyhessea, Peptococcus, Peptostreptococcus, Finegoldia, Porphyromonas, Corynebacterium, Alloscardovia, Mageeibacillus, Haemophilus, Duncaniella, Ralstonia, Sphingomonas, Pedobacter, Methylobacterium, Ruminococcus, Sediminibacterium, Pseudomonas, Aerococcus, Acinetobacter, Campylobacter, Stenotrophomonas, Sphingobacterium, Phyllobacterium* and others may be included in the microbial composition of the cervical canal. Dysbiotic disorders were more common in patients with HSIL. *Streptococcus* spp. always accompanied aerobic vaginitis (AV), whereas bacterial vaginosis (BV) was associated with the genera *Gardnerella, Fannyhessea, Prevotella, Dialister, Sneathia, Anaerococcus, Megasphaera, Finegoldia, Peptoniphilus, Porphyromonas, Parvimonas* and *Eubacterium* spp. When comparing the two methods, the genera *Peptoniphilus, Methylobacterium, Ralstonia spp, Sphingomonas, Phyllobacterium, Parvimonas, Anaerococcus* and *Ruminococcus* may be included in the microbial biomass in a significant proportion. Eubacterium spp. did not occur in the 16S sequencing method. Some representatives are detected together with each other in the RT-qPCR method (Femoflor - 16), e.g., *Sneathia* spp. + *Leptotrichia* spp. + *Fusobacterium* spp.; *Megasphaera* spp. + *Veillonella* spp. + *Dialister* spp.; *Mobiluncus* spp. + *Corynebacterium* spp., however, *Corynebacterium* spp., *Veillonella* spp., *Mobiluncus* spp., *Fusobacterium* spp., *Leptotrichia* spp. and *Peptostreptococcus* spp. were practically absent (or in a low percentage) in the microbiome of women with SIL.

## 1 Introduction

Currently, there is a global increase in cervical cancer (CC) incidence rates (Yuan et al., 2022; Qui et al., 2022). Incidence rates continue to rise, which necessitates identifying additional potential causes for human papillomavirus (HPV) persistence and the progression of squamous intraepithelial lesions (SIL) to CC (Liu et al., 2022). It is known that CC is etiologically linked to HPV, an oncogenic virus actively involved in the transformation of cervical epithelial cells (Lei et al., 2020). Over time, HPV infection can lead to the development of SIL, which may regress or progress to CC (Nieves-Ramírez et al., 2021). However, HPV can be eliminated in more than 90% of cases within 6–18 months, with viral persistence observed in 10% of women (Prilepskaya and Nazarova, 2014).

Recent data indicate that the cervicovaginal microbial community plays a significant role in HPV persistence (Li et al., 2020). A dysbiotic microbiome is associated with the development of cervical carcinogenesis (Gardella et al., 2022; Lin et al., 2022; Ntuli et al., 2022; Kaelin et al., 2022). Several concepts are related to the microbiota of the cervical canal and vagina. The microbiome is the totality of microorganisms and their genes that form a “second genome” in humans, providing ecological interactions among themselves and with the surrounding environment, thereby extending the genetic and functional capacities of the human genome. Microbiocenosis is an ecologically and spatially isolated part of the human microbiome, consisting of microorganisms from several genetically related types, linked by common environmental requirements, often significantly shaped by the community of microorganisms that comprise the microbiocenosis^1^. Numerous factors influence the microbiome and microbiocenosis composition, including socio-demographic, socio-economic (limited access to healthcare, poverty), epidemiological factors (multiple sexual partners, oral contraceptive use, hygiene habits, antibiotic intake, smoking), and ethnicity (Caucasian and Asian women show a higher prevalence of dominant *Lactobacillus* spp. microbiota compared to Hispanic and Black women) (Bowden et al., 2021; Bilgi et al., 2021; Pedroza-Gonzalez et al., 2022; Plisko et al., 2021; Wright et al., 2021).

Today, next-generation sequencing (NGS) methods based on the analysis of bacterial 16S rRNA genes allow an in-depth study of the structure of the cervical and vaginal microbial community to a level of detail that standard microbiological methods cannot achieve, while Femoflor-16 detects the DNA of 25 microorganisms. In recent years, NGS has expanded our understanding of the cervicovaginal microbiome composition. Studies on the microbial biomass of the female reproductive tract through 16S rRNA gene sequencing revealed that the vaginal microbiota of 110 healthy women of reproductive age contains 1010–1011 bacteria, predominantly lactobacilli (Chen et al., 2021). *Lactobacillus* spp. inhibit the colonization of the lower genital tract by other microorganisms through lactic acid, bacteriocins, and bio-surfactants, and also prevent HPV persistence (Kalia et al., 2020). Five vaginal community types were identified, dominated by *Lactobacillus crispatus* (*L. crispatus*) (CST I), *Lactobacillus iners (L. iners*) (CST II), *Lactobacillus gasseri (L. gasseri*) (CST III), and *Lactobacillus jensenii (L. jensenii*) (CST V). The CST IV community is a diverse group without specific dominant species, divided into subtypes: CST IV-A, which includes genera *Anaerococcus, Peptoniphilus, Corynebacterium, Prevotella, Finegoldia*, and *Streptococcus*, and CST IV-B, which includes genera *Atopobium, Gardnerella, Sneathia, Mobiluncus*, and *Megasphaera* (Molina et al., 2022; McClymont et al., 2022).

Recent data suggest that *L. crispatus, L. gasseri*, and *L. jensenii* protect against SIL progression in HPV infections as they produce a high quantity of D-isomer lactic acid, which increases vaginal mucus viscosity and enhances its ability to trap virions *(L. crispatus* and *L. gasseri* also produce a small amount of L-isomer). *L. iners* synthesizes the L-isomer of lactic acid and produces inerolysin, which creates pores in the vaginal epithelium, promoting HPV infection and persistence (Kalia et al., 2020; Nicolò et al., 2023; Xu et al., 2022). However, other studies show disagreements on the role of *L. iners*, with no noted association between CST II, microbial composition, and HPV infection (Wu et al., 2022).

Recently, special attention has been given to studying individual microorganisms in the development of SIL (Castanheira et al., 2021; Zhai et al., 2021). Some bacteria associated with the development of bacterial vaginosis (BV) and aerobic vaginitis (AV) have been linked to HPV-associated cervical diseases; however, most authors report conflicting results regarding the identified taxa (Li et al., 2022).

In our study, we examined genera and species of prokaryotic microorganisms using 16S rRNA gene sequencing method and analyzed cervix canal microbiocenosis data using RT-qPCR. A comparative analysis was conducted between microorganisms identified through NGS methods and RT-qPCR techniques.

## 2 Methods

### 2.1 Clinical material

From January 2022 to June 2023, a cervical examination was conducted among women of reproductive age at the Kulakov National Medical Research Center for Obstetrics, Gynecology, and Perinatology under the Ministry of Health of Russia. The examination included liquid/traditional cytology, real-time 21-type HPV testing, and extended colposcopy. A total of 152 patients with histologically confirmed SIL were included in the study. All participants signed informed consent to take part in the study. Pregnant and breastfeeding patients, as well as those who had undergone antibiotic therapy in the past 14 days, were excluded; reproductive age was defined as between 19 and 45 years.

The patients were divided into two groups according to the histological findings (with further division into two subgroups due to different research methods):

Group I – patients with histological diagnosis LSIL (*n* = 67). Subgroup Ia included (*n* = 31) patients with microbiome analysis performed using 16S rRNA gene sequencing, while Subgroup Ib (*n* = 36) had their microbiome analyzed by qPCR-RT.
Group II – patients with histological diagnosis HSIL (n=85). Subgroup IIa included (n=45) patients whose microbiome was analyzed by 16S rRNA gene sequencing, and Subgroup IIb (n=40) where microbiome analysis was performed by qPCR-RT.

All patients were surveyed about several clinical parameters, including HIV status. Survey results could be found in Supplementary Table 6.

### 2.2 Collecting the biomaterial for metagenomic study and RT-qPCR testing

Material for biocenosis analysis was collected into 1.5 mL Eppendorf tubes with 500 microliters of a 0.9% sodium chloride solution for HPV testing.

Swabs were collected into 1.5 mL tubes containing 300 microliters of 20 mM Tris–HCl buffer (pH = 7.2) for 16s rRNA gene sequencing and for RT-qPCR. The sterile disposable swab, sterile tubes and sterile 0.9% sodium chloride solution or 20 mM Tris–HCl buffer (pH = 7.2) were used for sample collection. The tubes with biomaterial were transported to laboratory. DNA isolation was performed in Laminar flow cabinet to exclude external and cross-sample contamination.

### 2.3 HPV

HPV testing was conducted using RT-qPCR with the “HPVquant-21” reagent kit (DNA Technology, Russia), which detects, genotypes, and identifies HPV viral load for 21 types (6, 11, 16, 18, 26, 31, 33, 35, 39, 44(55), 45, 51, 52, 53, 56, 58, 59, 66, 68, 73, 82).

### 2.4 RT-qPCR (Femoflor-16 test)

The cervical canal microbiocenosis was studied using RT-qPCR with Femoflor reagents (a PCR amplification mix specific to all bacteria for determining total bacterial load, as well as mixes specific to lactobacilli and opportunistic microorganisms). RT-qPCR was conducted on an amplifier with real-time detection using the DT-96. When evaluating the cervical biocenosis, total bacterial load was assessed, along with the presence of lactobacilli, obligate anaerobic and facultative anaerobic microorganisms, yeast-like fungi, Ureaplasma spp., Mycoplasma hominis, and Mycoplasma genitalium. The quantity of lactobacilli was calculated relative to the total bacterial load as the logarithm of the ratio. The resulting data were divided by percentage of lactobacilli: share > 80% (healthy state), 20–80% (moderate dysbiosis), and 0–20% (severe dysbiosis).

### 2.5 DNA isolation and library preparation for sequencing

DNA was isolated from the biomaterial using the DNeasy Blood and Tissue kit (Qiagen, United States) following the manufacturer's instructions. Quality control of the isolated prokaryotic DNA was performed by qPCR using the primers recognizing the V4 DNA region encoding 16S rRNA (515F and 806R).

The libraries were prepared for sequencing in two steps. At the first step, the V4 region of 16S rRNA was amplified using the primers to the prokaryotic V4 DNA region encoding 16S rRNA (515F and 806R) containing technical sequences for the MGI adapters. At the second step, amplification was performed using primers containing unique barcodes and primer technical sequences. The concentrations of prepared libraries were measured by Qubit Flex (Life Techonologies, United States) using dsDNA HS Assay Kit (Life Technologies, United States) following the manufacturer's protocol. The quality of the prepared libraries was assessed using Bioanalyzer 2,100 with the High Sensitivity DNA kit (Agilent Technologies) according to the manufacturer's instructions. After the libraries were circularized and sequenced in the paired-end mode using the DNBSEQG-400 platform with the DNBSEQ-G400RS High-throughput Sequencing Set PE150 kit according to manufacturer's protocol (MGI Tech). FastQ files were generated using the zebracallV2 software by the manufacturer (MGI Tech).

### 2.6 Sequencing data processing

The obtained overlapping paired reads were merged into unified nucleotide sequences and grouped based on the sequence identity and possible polymerase errors using Qiime2 v.2022.8. Each sequence group was assigned with the taxonomic class (family and genus) using the RDP classificator. Furthermore, each sequence group was aligned using blast v2.13.0 with default settings against the 16S rRNA database followed by determining the species in a read group. Sequence groups with the content of <0.01% in the sample were excluded from the analysis.

### 2.7 Statistical analysis

For the qualitative assessment of the bacterial composition, only those samples with a share of ≥1% in at least two patients were included in the study. Quantitative assessment of microorganisms included a share of at least 5% in at least two patients. Statistically significant deviations in quantitative assessment were evaluated using the Wilcoxon signed-rank test. Statistically significant deviations in qualitative assessment were evaluated using the Chi-squared test or Exact Fisher's test. Biodiversity assessment was performed using the Shannon and Simpson indices. Differences were considered statistically significant at p <0.05. Multiple comparison correction was performed by Bonferroni method.

## 3 Results

### 3.1 Clinical and demographic characteristic of samples

A total of 152 patients with histologically confirmed SIL were included in the study. Group II patients had higher age (about 3 year) and lower age of sexual initiation (about half-year). Difference was statistically significant (Supplementary Table 1). It was noted that the cytological diagnosis of negative for intraepithelial lesion or malignancy (NILM) was statistically more frequent in patients with a histological diagnosis of LSIL in 20 cases (30%) compared to the HSIL group in 7 cases (8%). Additionally, among patients with a cytological diagnosis of ASCUS, 15 had a confirmed SIL diagnosis.

Patients in the Group I used hormonal contraception about two times more frequently than in the Group II, but difference was not statistically significant. In another rank features statistically significant differences were not found as well (Supplementary Table 2). Patients in the Group II had 1.5 times more likely to be pregnant and had less endometrial/cervical polyps compared Group I. No other statistically significant differences were found. Both studied groups had only one HIV positive patients (Supplementary Table 3).

### 3.2 HPV types

**Figure 1:**
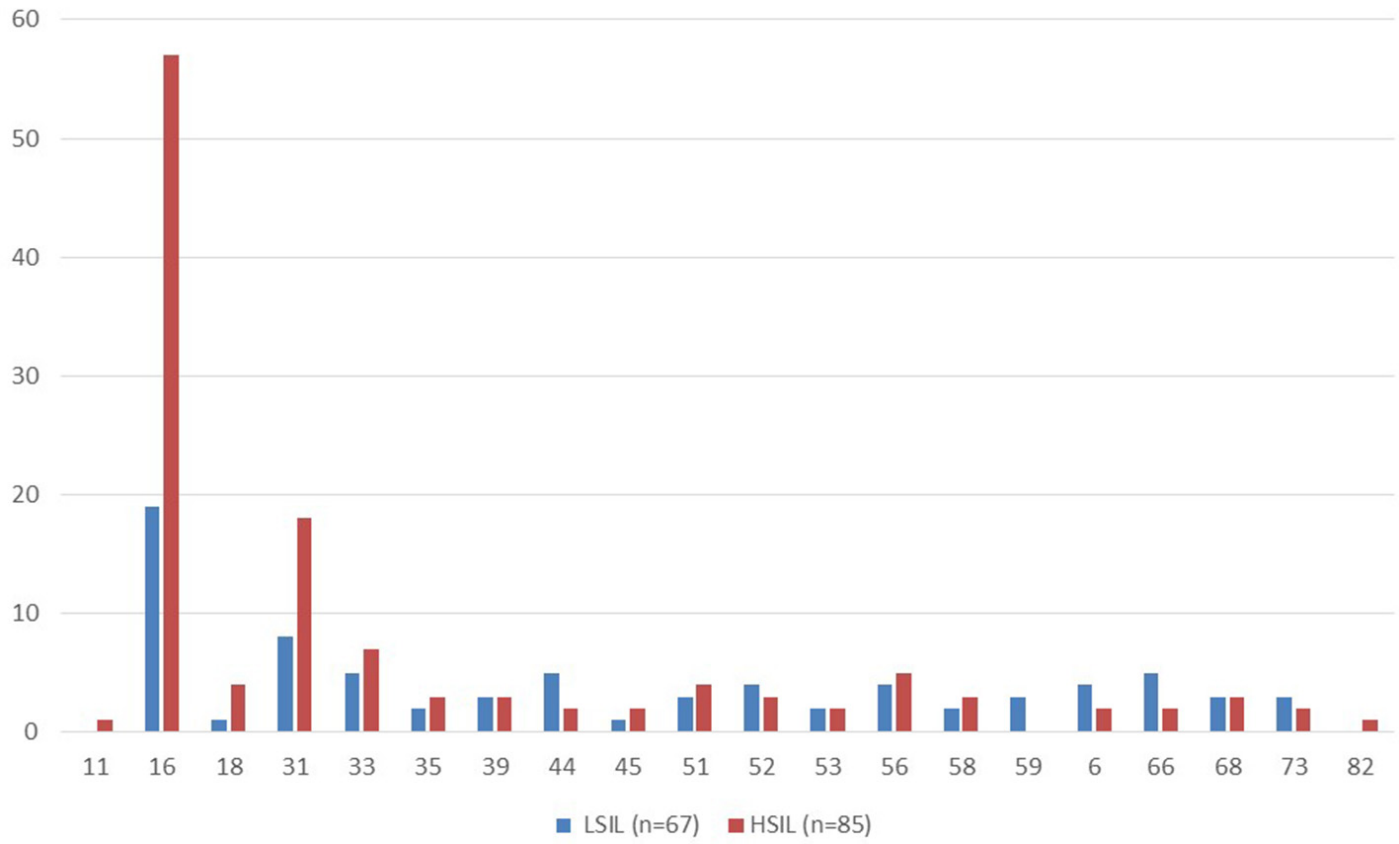
Distribution of HPV types in the studied groups.

The HSIL group had a higher proportion of patients with at least one type of HPV detected (98%) compared to the LSIL group, where HPV was present in 70% of cases (*p* < 0.05). The most common HPV type was 16, detected in 57 out of 85 (67%) patients with HSIL and in 19 out of 67 (28%) with LSIL. The second most common type was type 31, found in 18 out of 85 (21%) HSIL patients and in 8 out of 67 (12%) with LSIL. Types 33 and 56 were the third and fourth most common. Types 82 and 11 were rarely detected (Figure 1).

### 3.3 Evaluation of microbial diversity

Microbial diversity was estimated only for groups Ia and IIa. It was higher in HSIL group about 40 percent on average. But no statistically significant differences in diversity were found between groups (*p* > 0.05) (Table 1).

**Table 1:** Assessment of microbial diversity in the study groups. Data are presented as median and interquartile range (Q25-Q75).

**Diversity index**	**Group Ia**	**Group IIa**	**P-value**
Shannon	0.61 (0.39–1.01)	0.80 (0.24–1.15)	0.93
Simpson	0.32 (0.19–0.49)	0.38 (0.10–0.61)	0.88

### 3.4 Cervical microbiocenosis in patients with SIL

In the study, the cervical canal microbiocenosis was examined using PCR-RT in 36 patients with LSIL and 40 with HSIL.

Our results showed that in the LSIL group, 7 out of 36 patients (19.4%) had absolute normocenosis, 12 out of 36 (33.3%) had conditional normocenosis (including vulvovaginal candidiasis and Ureaplasma infection), and 17 out of 36 (47.2%) had various types of dysbiosis (Table 2). The proportion of anaerobic vaginitis was 8 out of 17, with 3 out of 8 classified as moderate and 5 out of 8 as severe. AV accounted for 5 out of 17 dysbiosis cases, with 1 out of 5 classified as moderate and 4 out of 5 as severe. Mixed dysbiosis represented 4 out of 17 cases, with 3 out of 4 classified as moderate and 1 out of 4 as severe.

**Table 2:** Prevalence of various dysbiosis types in patients with LSIL and HSIL detected by Femoflor-16.

**№**	**Number of dysbioses cases**	**Anaerobic**		**Aerobic**		**Mixed**	
		**Moderate**	**Apparent**	**Moderate**	**Apparent**	**Moderate**	**Apparent**
LSIL (*n* = 36)	17 (47.2 %)	3 (8.3%)	5 (13.9%)	1 (2.8%)	4 (11.1%)	3 (8.03%)	1 (2.8%)
HSIL (*n* = 40)	21 (52.5%)	6 (15%)	7 (17.5%)	3 (7.5%)	1 (2.5%)	1 (2.5%)	3 (7.5%)

A similar analysis was conducted for the 40 patients in the HSIL group. It was found that absolute normocenosis was present in 6 out of 40 patients (15%), conditional normocenosis in 13 out of 40 (32.5%), and dysbiosis in 21 out of 40 (52.5%) cases. Notably, anaerobic vaginitis accounted for 13 out of 21 cases, with 6 out of 13 classifieds as moderate and 7 out of 13 as severe. AV was observed in 4 out of 21 patients, with 3 out of 4 cases classified as moderate and 1 out of 4 as severe. Among the remaining dysbiosis cases, mixed dysbiosis accounted for 4 out of 21, with 1 out of 4 classified as moderate and 3 out of 4 as severe (Table 2).

BV was mainly associated with the following microorganisms or their complexes: *Gardnerella vaginalis* + *Prevotella bivia* + *Porphyromonas spp*. and *Eubacterium spp*., while *Streptococcus spp*. and *Enterobacterium* spp. were pathogens identified for AV (Table 3).

**Table 3:** Prevalence of microorganisms (in the highest percentage) in patients with dysbiosis in Femoflor 16.

**Microorganism types contributing to dysbiosis development**	**Representatives of anaerobic dysbiosis**		**Representatives of aerobic dysbiosis**		**Representatives of mixed dysbiosis**	
	**LSIL (*n* = 8)**	**HSIL (*n* = 13)**	**LSIL (*n* = 5)**	**HSIL (*n* = 4)**	**LSIL (*n* = 4)**	**HSIL (*n* = 4)**
*Gardnerella vaginalis+ Prevotella bivia+Porphyromonas* spp.	7	13	-	-	3	4
*Eubacterium* spp.	5	9	-	-	3	2
*Veilonella* spp*.+Megasphaera* spp*.+Dialister* spp.	3	1	-	-	2	2
*Atopobium vaginae*	3	1	-	-	-	1
*Lachnobacterium* spp*.+ Clostridium* spp.	1	1	-	-	1	1
*Peptostreptococcus* spp.	*1*	*3*	*-*	*-*	*1*	*1*
*Mobiluncus spp.+ Corynebacterium* spp.	*-*	*1*	*-*	*-*	*2*	*2*
*Sneathia* spp*.+ Leptotrihia* spp*.+Fusobacterium* spp.	*-*	*1*	*-*	*-*	*1*	*2*
*Streptococcus* spp.	*-*	*-*	*5*	*4*	*1*	*4*
*Enterobacterium* spp.	*-*	*-*	*-*	*1*	*3*	*-*
*Staphylococcus* spp.	*-*	*-*	*-*	*-*	*1*	*-*

No statistically significant differences in dysbiosis prevalence were found between the LSIL and HSIL groups (*p* > 0.05).

No statistically significant differences in the prevalence of various microorganisms and their complexes were found in patients with SIL and dysbiosis (*p* > 0.05).

### 3.5 Qualitative and guantitative assessment of non-*Lactobacillus* microorganisms in patients with SIL using 16S rRNA gene sequencing

Next-generation sequencing identified over 100 genera of prokaryotic microorganisms, with 43 being the most prevalent (each comprising at least 1% of reads in two or more samples). The non-*Lactobacillus* microbiome of the cervical canal was studied in patients with LSIL (*n* = 31) and HSIL (*n* = 45). Qualitative analysis revealed statistically significant differences: *Fannyhessea vaginae* (formerly *Atopobium vaginae*) was more frequently detected in the HSIL group (*p* < 0.05), while *Pseudomonas lini, Escherichia/Shigella spp*., and *Ureaplasma spp*. were more commonly found in the LSIL group (*p* < 0.05) (Table 4). Quantitative analysis showed that the proportion of *Fannyhessea vaginae* was significantly higher in the HSIL group, whereas *Ureaplasma spp*. were more prevalent in the LSIL group (*p* < 0.05).

**Table 4:** Qualitative and quantitative characteristics of the content of statistically significant taxa in patients with SIL.

**Genus**	**Data type**	**LSIL (*n* = 31)**	**HSIL (*n* = 45)**	***P*-value**	**Adjusted *p*-value**
Fannyhessea	*n* (percent)	3 (9.7%)	13 (28.9%)	0.05	1
	Median (Q25–Q75)	0 (0–0)	0 (0–9.59)	0.03	1
Pseudomonas	*n* (percent)	14 (45.2%)	8 (17.8%)	0.02	0.853
	Median (Q25–Q75)	-	-	-	
Escherichia/Shigella	*n* (percent)	9 (29%)	4 (8.9%)	0.03	1
	Median (Q25–Q75)	-	-	-	
Ureaplasma	*n* (percent)	15 (48.4%)	8 (17.8%)	0.009	0,4
	Median (Q25–Q75)	0 (0–7.37)	0 (0–2.85)	0.005	1

The data are presented as the number of patients in the group in whom the specified genus of microorganism was detected (in brackets, the percentage of the total number of patients in the group). *P*-value Quantitative metrics are presented as the median and interquartile range. *P*-value—Kruskal-Wallis test.

It's noteworthy that the microbiome in patients with bacterial vaginosis (BV) is associated with the following anaerobic genera and species: *Gardnerella vaginalis, Fannyhessea vaginae, Dialister micraerophilus, Sneathia sanguinegens, Anaerococcus, Megasphae ramassiliensis, Prevotella, Finegoldia magna, Peptoniphilus, Porphyromonas*, and *Parvimonas micra*. In contrast, AV is predominantly associated with the genus *Streptococcus*.

The study also identified “unusual” microorganisms (Table 5).

**Table 5:** The ≪Unusual≫representatives that were found in the study.

**Genus**	**Major representatives (species)**
*Duncaniella*	-
*Sediminibacterium*	-
*Mageeibacillus*	-
*Methylobacterium*	*organophilum*
*Stenotrophomonas*	*maltophilia*
*Haemophilus*	*-*
*Phyllobacterium*	*myrsinacearum, endophyticum, bourgognense, brassicacearum*
*Pedobacter*	*nutrimenti, steynii*
*Acinetobacter*	*baumannii*
*Ralstonia*	*syzygii, pickettii*
*Finegoldia*	*magna*
*Limosilactobacillus*	*antri, coleohominis, reuteri, mucosae*
*Ruminococcus*	*-*
*Campylobacter*	*hominis, ureolyticus, showae*
*Aerococcus*	*viridans*
*Alloscardovia*	*omnicolens*
*Sphingobacterium*	*spiritivorum*
*Sphingomonas*	*alpina, oligophenolica, echinoides*

### 3.6 Predominant *Lactobacillus* species in patients with SIL under normocenosis, moderate, and severe dysbiosis (anaerobic, aerobic, and mixed) identified by 16S sequencing

This study examined the prevalence of normocenosis and various types of dysbiosis, as well as determined the dominant *Lactobacillus* species in these conditions among 76 patients with SIL (Table 6).

**Table 6:** Dominant *Lactobacillus* species by microbiome status.

**Group**	**Condition**	***n* (%)**	**Dominant *Lactobacillus* species**	***n* dominant species (%)**
LSIL (*n* = 31)	Normocenosis	19 (61.3%)	*L. crispatus*	10 (52.6%)
	Moderate dysbiosis	8 (25.8%)	*L. crispatus*	5 (62.5%)
	Apparent dysbiosis	4 (12.9%)	*L. crispatus*	2 (50%)
HSIL (*n* = 45)	Normocenosis	25 (55.5%)	*L. iners*	14 (56%)
	Moderate dysbiosis	10 (22.2%)	*L. gasseri*	6 (60%)
	Apparent dysbiosis	10 (22.2%)	*L. iners L. crispatus*	4 (40%) 4 (40%)

In the LSIL group (*n* = 31), normocenosis was detected in 19 out of 31 patients (61.3%), while dysbiosis was present in 12 out of 31 (38.7%). Anaerobic dysbiosis were in 6 out of 12 patients, with 5 out of 12 cases being moderate and 1 out of 12 severe. Aerobic dysbiosis was observed in 3 out of 12 cases, with 2 out of 3 being moderate and 1 out of 3 severe. Mixed dysbiosis was also noted in 3 out of 12 patients, with 1 out of 3 being moderate and 2 out of 3 severe.

In the HSIL group (*n* = 45), normocenosis was found in 25 out of 45 patients (55.5%). Dysbiosis was present in 20 out of 45 (44.5%). Anaerobic dysbiosis, similar to the LSIL group, was the most common, occurring in 13 out of 20 patients, with 6 out of 13 cases being moderate and 7 out of 13 severe. Aerobic dysbiosis was identified in only 2 out of 20 patients (one moderate and one severe case). Mixed dysbiosis was slightly more frequent, observed in 5 out of 20 patients, with 3 out of 5 being moderate and 2 out of 5 severe.

In cases of BV, the predominant genera were *Gardnerella, Fannyhessea, Dialister, Sneathia, Anaerococcus, Megasphaera, Prevotella, Finegoldia, Peptoniphilus, Porphyromonas*, and *Parvimonas*. In contrast, AV was primarily associated with the genus *Streptococcus*.

The Table 6 illustrates the prevalence of dominant *Lactobacillus* species in patients with LSIL and HSIL across different microbiome conditions. In the LSIL group, *L. crispatus* was the predominant species across all conditions. In the HSIL group, *L. iners* was most common in normocenosis and severe dysbiosis, while *L. gasseri* dominated in moderate dysbiosis (Table 6).

## 4 Discussion

The analysis of the conducted study revealed differences in the occurrence of various microorganisms identified by qPCR-RT and 16S rRNA gene sequencing methods. It was found that the genera *Peptoniphilus, Methylobacterium, Ralstonia, Sphingomonas, Phyllobacterium, Parvimonas, Anaerococcus*, and *Ruminococcus* may occupy a significant portion of the cervical canal microbiome. *Eubacterium* spp. was not detected using the 16S rRNA gene sequencing method. Bacteria indicated in the qPCR-RT method, such as *Peptostreptococcus* spp., *Corynebacterium* spp., *Veillonella* spp., *Mobiluncus* spp., *Fusobacterium* spp., and *Leptotrichia* spp., were almost absent in patients when analyzed using the 16S rRNA gene sequencing method. It could be due to high sensitivity of qPCR-RT compared to bulk 16S rRNA gene sequencing method.

In the study of microbiocenosis, the main share of bacteria associated with BV included *Gardnerella vaginalis* + *Prevotella bivia* + *Porphyromonas* spp. and *Eubacterium* spp., while in the microbiome analysis, *Gardnerella vaginalis, Fannyhessea vaginae, Prevotella, Porphyromonas, Dialister micraerophilus, Sneathia sanguinegens, Anaerococcus, Megasphaera massiliensis, Finegoldia magna, Peptoniphilus*, and *Parvimonas micra* were identified. In both methods, *Streptococcus* spp. was the only representative of AV. Studies by Wei B, Wu S, and Wu M have shown that the genera *Gardnerella, Fannyhessea, Dialister, Sneathia, Prevotella, Porphyromonas, Megasphaera, Anaerococcus*, and *Streptococcus* can contribute to HPV infection persistence and lead to the development of SIL, consistent with our findings (Wu et al., 2022; Wei et al., 2022; Wu et al., 2021; Mitra et al., 2015). According to Russian authors, among patients with HPV-/HPV+ and SIL, qPCR-RT detected *Gardnerella vaginalis* in association with *Eubacterium* spp., *Veillonella* spp. + *Megasphaera* spp. + *Dialister* spp., and *Atopobium vaginae* among anaerobic microorganisms, which aligns with our results (Kononova et al., 2015)^2^. The main limitation of the study is separate set of samples for each type of methods.

Comparing both methods, we found that in the group of patients with LSIL, the frequency of dysbiosis determined by qPCR-RT was 47.2%, while by 16S rRNA gene sequencing it was 38.7%. In the group of patients with HSIL, the frequency of dysbiosis was 52.5% when studying the biocenosis and 44.5% when analyzing the microbiome. Thus, dysbiosis was more frequently detected in patients with HSIL. Our findings are consistent with current data and support existing observations of a higher frequency of dysbiosis in patients with severe epithelial lesions (Tosado-Rodríguez et al., 2023).

Regarding different types of lactobacilli, *L. crispatus* dominated in the group of patients with LSIL in cases of normocenosis and all types of cervical dysbiosis. In patients with HSIL, *L. iners* predominated in normocenosis, while *L. gasseri, L. iners*, and *L. crispatus* were more common in various forms of dysbiosis. A 2016 study demonstrated that *L. iners* more frequently dominates in cases of HSIL (Xu et al., 2022; Piyathilake et al., 2016).

It is worth noting a 2023 article by Russian authors from Novosibirsk, in which the microbial landscape of women with cytological conclusions of NILM (healthy contol), cytological/histological conclusions of SIL, confirmed CC, and patients post-CC treatment (radiation, chemoradiotherapy, surgical, and combined treatment) was analyzed using NGS and PCR (Ivanov et al., 2023). The study observed that increased diversity of the cervicovaginal microbiome correlates with the severity of SIL lesions, including in patients post-CC treatment. According to several authors, the use of radiation and chemotherapy in gynecological oncology, as well as the presence of SIL, may increase the biodiversity of the cervicovaginal microbiome, which is supported by findings in other studies (Tsakmaklis et al., 2020). In our study, only groups with histological diagnoses of LSIL and HSIL were included, in contrast to our colleagues' study, which, despite having a wide range of groups, had a small sample size in the LSIL group (24 patients, with not all having undergone histological examination), HSIL group (22 patients), and CC group (17 patients), while the majority of the study sample comprised patients post-CC treatment (101). The 2023 article noted a decline in lactobacilli dominance from NILM to CC, consistent with our findings and those of other authors (Guo et al., 2022). Additionally, *L. iners* was particularly prevalent in the group after chemoradiotherapy for CC and HSIL, while *L. gasseri* predominated in the CC group. In our study, these species of lactobacilli dominated in the HSIL group, aligning with results from Mexican authors in 2021 (Nieves-Ramírez et al., 2021). However, in our study, *L. crispatus* consistently dominated in the LSIL group in cases of normocenosis and various dysbiosis types. Both studies show that most samples with SIL were enriched with microorganisms typical for BV and AB. These organisms were more frequently observed with increasing severity of cervical lesions, consistent with our findings, where dysbiosis prevalence in patients with HSIL was higher than in patients with LSIL. The main microbial representatives found in the 2023 study almost align with the results of our study, which is corroborated by other studies (Kang et al., 2021; Li et al., 2023). In the Novosibirsk study, patients were tested for only 12 types of HPV, with type 16 being the most common in both studies, in agreement with other research findings (Zhang et al., 2020; Bräutigam et al., 2022; Karadža et al., 2021; Jin et al., 2021).

Both method could be successfully apply to estimate microbiome status of cervical canal. The qPCR-RT is more sensitive method, but the determining of *Lactobacillus species* must be added. Because it could influence on SIL status.

## Data Availability

The data presented in the study are deposited in the NCBI BioProject repository, accession number PRJNA1041987.

## Footnotes

^1^Clinical guidelines for the diagnosis and treatment of diseases accompanied by pathological discharge from the female genital tract.

^2^Бактериальный вагиноз как кофактор развития интраэпителиальных неоплазий.
